# When Kids Radiate: Low-Resolution Thermography for Total Energy Expenditure Estimation in Pediatric Patients - A Proof of Concept

**DOI:** 10.1109/OJEMB.2026.3667036

**Published:** 2026-02-23

**Authors:** Sofiane Beloucif, Mario Francisco Munoz, Kevin Albert, Louise Faineant, Rita Noumeir, Philippe Jouvet

**Affiliations:** Faculty of Science, Department of Computer ScienceUniversité de Sherbrooke 7321 Sherbrooke QC J1K 2R1 Canada; CHU Sainte-Justine Research Center Montreal QC H3C 1K3 Canada; CHU Sainte-Justine Research Center, CHU Sainte-Justine Hospital Montreal QC H3C 1K3 Canada; CHU Sainte-Justine Research Center, CHU Sainte-Justine Hospital Montreal QC H3T 1C5 Canada; University of Montreal Montreal QC H3C 1K3 Canada; École de Technologie Supérieure 514734 Montreal QC H3C 1K3 Canada; CHU Sainte-Justine Research Center, CHU Sainte-Justine Hospital Montreal QC H3T 1C5 Canada; Biomedical Information Processing Lab, École de Technologie SupérieureUniversité du Québec 14845 Montreal QC H3C 1K3 Canada; CHU Sainte-Justine Research Center, CHU Sainte-Justine Hospital Montreal QC H3T 1C5 Canada; University of Montreal Montreal QC H3C 1K3 Canada

**Keywords:** Computer vision, infrared thermography, pediatrics intensive care, remote patient monitoring (RPM), segmentation

## Abstract

*Goal:* Remote metabolic monitoring is a growing field in pediatric care, aiming to reduce invasive procedures while ensuring continuous assessment. However, clinical adoption remains limited by occlusions, poor image quality, and the scarcity of annotated data. In this study, we propose a framework based on deep learning to estimate total energy expenditure (TEE) in pediatric patients using low-resolution thermography. Our pipeline uses a UNet segmentation model trained to isolate anatomically relevant regions despite visual noise and occlusions. Radiative heat transfer computations are then applied to derive energy expenditure metrics. We tested our method in a cohort of 116 pediatric patients, achieving a mean TEE of 1547 kcal/m$^{2}$/day and a mean absolute error of 279 kcal/m$^{2}$/day. These results highlight the feasibility of thermography as a noninvasive, scalable alternative for metabolic monitoring in Pediatric Intensive Care Units (PICUs), especially in data-constrained environments.

## I. Introduction

Remote patient monitoring (RPM) [1] of metabolic activity is an essential component of modern pediatric care, offering non-invasive alternatives to traditional methods such as indirect calorimetry. In pediatric intensive care units (PICUs), the continuous assessment of total energy expenditure (TEE) can inform clinical decisions about nutrition, recovery, and treatment efficacy. However, current gold standard methods remain invasive, technically demanding, and impractical for routine bedside use.

Thermal imaging has gained attention as a contactless, spatio-temporal modality capable of capturing skin temperature variations. Previous studies have demonstrated the correlation between surface temperature and metabolic heat loss, suggesting that thermography could support real-time monitoring of energy expenditure in newborns and children. In particular, infrared thermography has been shown to align closely with calorimetric measurements in controlled settings [2]. Studies such as [3] have also adapted thermography for pediatric use in resource-limited environments, however, with limited automation.

Despite its potential, several challenges hinder the integration of thermal imaging into clinical workflows. Thermal images are often low resolution, especially when captured using cost-effective hardware. The acquisition of thermographic data can be challenging in pediatric patients due to their small body size, frequent movement, and the presence of occlusions such as ventilation tubes, clothing, or medical equipment. These factors complicate pose estimation and energy modeling. Moreover, deep learning-based approaches typically require large, balanced datasets, which are difficult to obtain in hospital environments due to ethical constraints and data annotation costs.

To overcome these PICU-related barriers in high-resolution color imaging, recent work by Munoz et al. [4] has shown that combining deep convolutional networks (e.g., DeepLabV3+) with transformer-based foundation models (e.g., SAM) can enhance segmentation robustness in cluttered clinical environments, however, these methods remain unavailable in thermography due to limited spatial resolution. Additional efforts have also addressed the thermal modeling aspects, incorporating parameters such as stable skin emissivity [5] and garment insulation [6], both of which are critical to accurately compute radiative energy transfer. In this work, we propose a novel deep learning pipeline to estimate total energy expenditure in pediatric patients using low-resolution thermal imaging. Our approach integrates image segmentation, temperature analysis, and radiative modeling to provide a fully automated, non-invasive estimation of metabolic activity suitable for deployment in PICU settings.

Our key contributions are as follows:

• *Dataset:* We curated and manually annotated a dataset of 102 thermal images from pediatric patients. This annotated dataset was used exclusively for training and validating the segmentation model, as it provides ground-truth masks for supervised learning.
• *Segmentation Model:* We trained a UNet-based segmentation model tailored to pediatric anatomy and thermographic noise, enabling reliable isolation of relevant body regions.
• *TEE Estimation Framework:* We implemented a radiative heat transfer model to compute energy expenditure based on segmented temperature maps, and tested it on a cohort of 116 patients. This second dataset was not manually annotated and is independent from the 102-image segmentation dataset.
• *THERMIS Application:* We developed THERMIS (*Thermal Health Evaluation and Research on Metabolic via Image Segmentation*), a desktop application integrating dataset processing, segmentation, and TEE estimation into a user-friendly interface adapted to the needs of French-speaking clinical staff.

With this work, we aim to advance the integration of thermal imaging and deep learning in pediatric metabolic monitoring, offering a practical tool for real-world clinical deployment.

## II. Materials and Methods

This section describes the data acquisition process, manual annotation strategy, segmentation architecture, heat-based clustering method, and physical modeling used for estimating TEE. Fis. 3 illustrates the end-to-end pipeline.

**Fig. 1.**
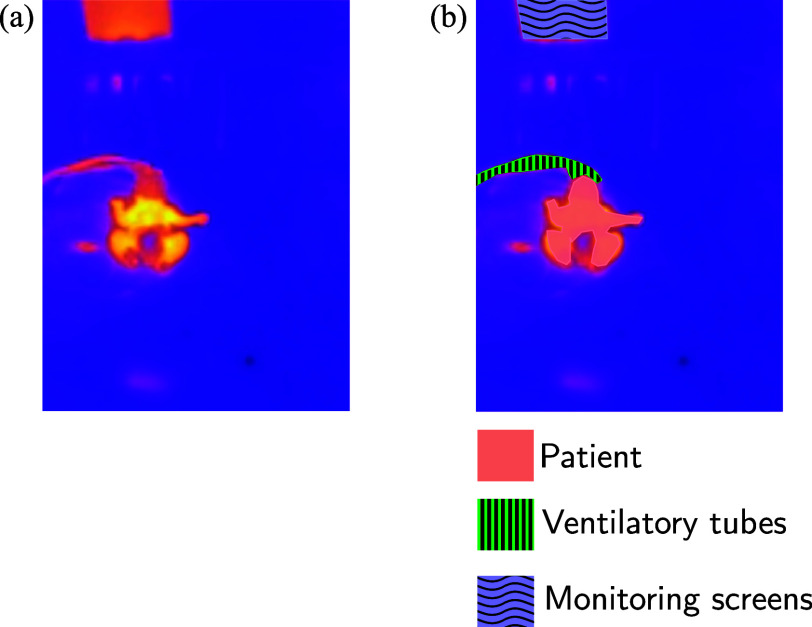
Comparison of thermal images before and after manual annotation. (a) Original thermal image. (b) Annotated version highlighting the three semantic classes: patient (peach), ventilatory tubes (green striped), and monitoring screens (blue wavy).

**Fig. 2.**
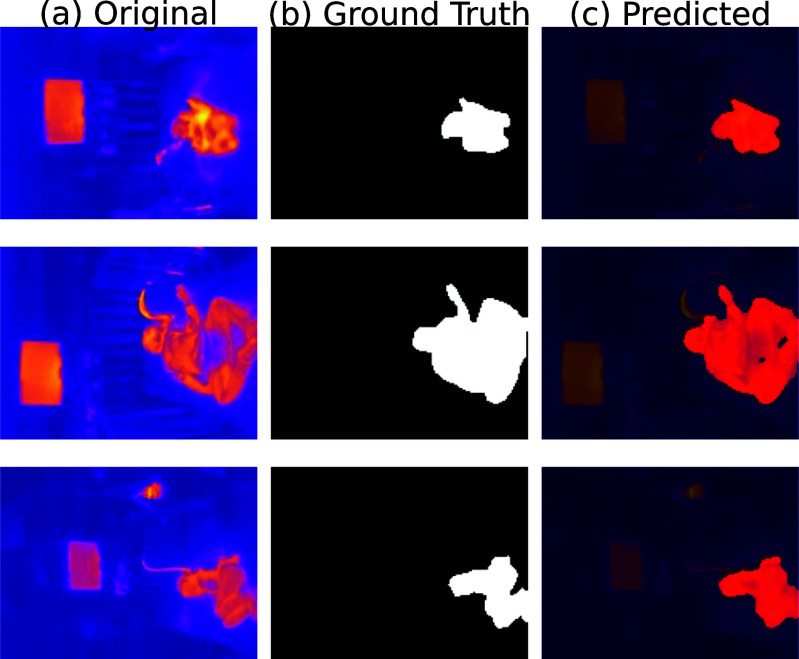
Representative segmentation examples from the test set, showing the original thermal image (left), the ground-truth mask (middle), and the predicted segmentation overlaid (right).

**Fig. 3.**
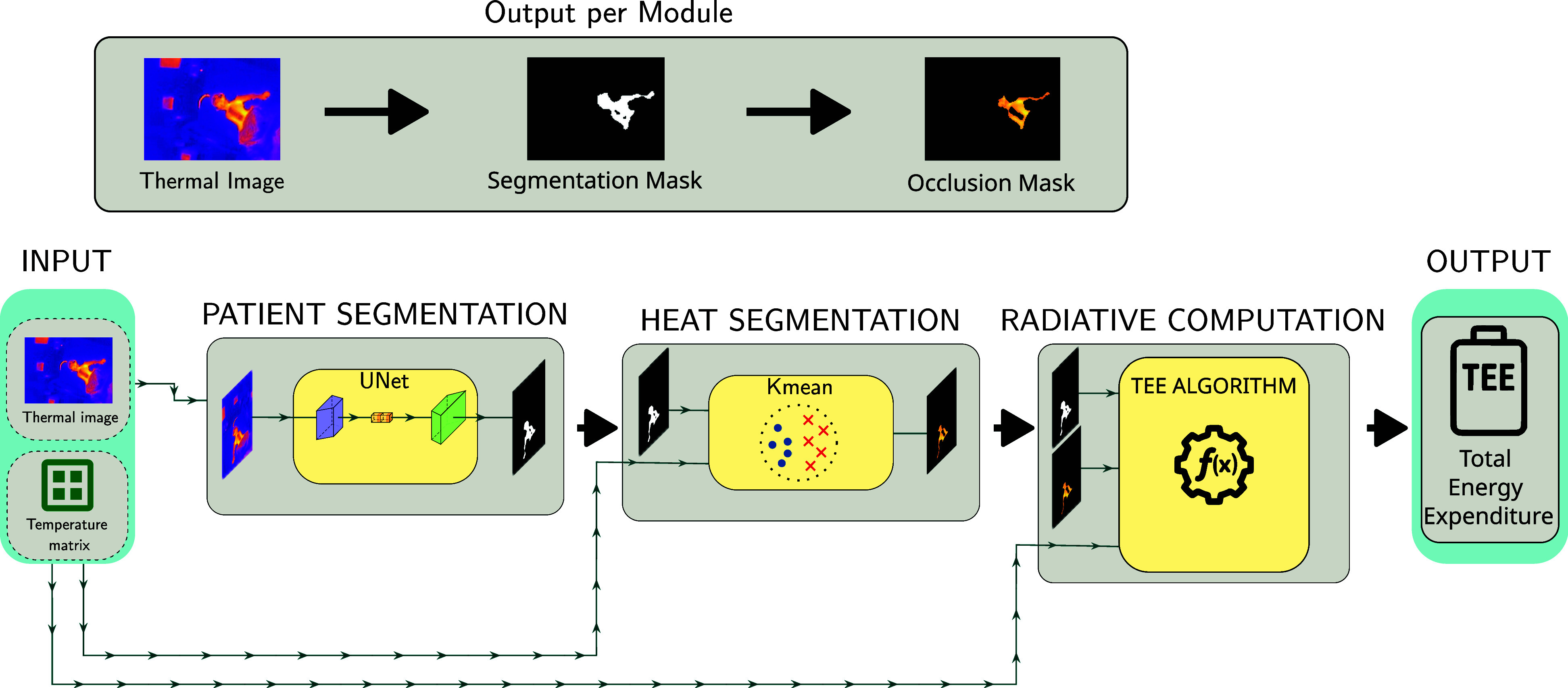
Overview of the proposed pipeline for TEE estimation. From left to right: acquisition of thermal images and corresponding temperature matrices; patient segmentation using a UNet with ResNet50 backbone; heat segmentation via Kmeans clustering to detect bare-skin regions and occlusions; followed by radiative modeling to ultimately compute the TEE.

### A. Thermal Image Acquisition

Thermal images were collected using a FLIR Lepton long-wave infrared (LWIR) thermography camera in a PICU setting. Each image has a spatial resolution of $160\times 120$ pixels and is accompanied by a temperature matrix capturing absolute surface temperatures (in $^\circ$C) per pixel.

The Lepton module was operated in radiometric T-linear mode, which applies the factory calibration (automatic non-uniformity correction and radiometric temperature conversion). The Lepton module was operated in radiometric T-linear mode, which applies the factory calibration (automatic non-uniformity correction and radiometric temperature conversion). As the clinical infrared images database was initially not designed to have the absolute thermal value. No other calibration were performed.

All acquisitions were performed after parent consent was obtained and stored in a secured database which contained more than 500 patients data currently (ethical committee approval protocol number 2016-1242) [7].

### B. Manual Annotation and Dataset Preparation

To enable semantic segmentation, a dataset of 102 thermal images was manually annotated using three classes: patient, ventilatory tubes, and monitoring screens. These categories were selected as they represent the most common sources of occlusion or thermal activity in intensive care environments. Manual polygon annotations were used to create accurate ground truth masks for supervised learning. Fig. 1 illustrates an example of the raw thermal image and its corresponding annotated version, where each semantic class is visually differentiated.

### C. Semantic Segmentation Model

We employed a UNet architecture with a ResNet-50 encoder backbone to segment patients from the background. UNet’s encoder–decoder structure, combined with skip connections, facilitates effective feature propagation and preserves spatial resolution—crucial for thermal analysis. To enhance generalization, we applied the following data augmentation techniques: random flipping (horizontal and vertical), rotation, scaling, brightness and contrast adjustments, Gaussian noise injection, and normalization. The model was trained using categorical cross-entropy loss, optimized with Adaptive Moment Estimation (Adam), and early stopping was applied based on validation performance.

To further evaluate the robustness of our approach, we compared UNet with the more recent DeepLabV3 architecture, also employing a ResNet-50 backbone. For both architectures, the learning rate (lr) and batch size (bs) were systematically varied to determine the optimal configuration.

Among the various training configurations (Table I), the optimal UNet model slightly outperformed DeepLabV3 in terms of Mean IoU and Dice coefficient, although DeepLabV3 achieved a marginally higher Pixel Accuracy.

**TABLE I:** Segmentation Performance Metrics of the Best Models From Each Architecture

**Metric**	**UNet**	**DeepLabV3**
Mean IoU	0.753	0.748
Mean Dice coefficient	0.843	0.837
Pixel Accuracy	0.984	0.986

#### 1) Evaluation Metrics

The models were evaluated on a separate test set of 80 annotated thermal images, distinct from the training set and chosen at random. An optimal activation threshold was selected to maximize the Intersection-over-Union (IoU) score. Table I summarizes the performance metrics for the best model from each architecture.

Although the similar performance, UNet was selected as the final segmentation model because its simpler architecture requires fewer parameters, making it easier to train and fine-tune on smaller datasets.

These findings are consistent with prior studies showing that UNet performs remarkably well even with limited datasets [8], whereas DeepLab models often exhibit suboptimal performance on smaller datasets [9]. The full results of all tested configurations are provided in Appendix.

#### 2) Qualitative Observations

As shown in Fig. 2, the segmentation model demonstrated strong qualitative performance across a range of challenging scenarios. Each row in the figure presents a distinct patient case, illustrating the model’s ability to generalize across both anatomical and environmental variability.

For instance, the first row corresponds to a newborn with a small body surface area and limited thermal contrast, yet the model accurately identifies the patient region without including surrounding artifacts. The second and third rows illustrate older children in different positions, including one curled-up posture where limbs overlap and the torso is partially occluded. Despite these complex geometries, the predicted masks remain coherent and well-aligned with the ground truth.

These qualitative observations are supported by the quantitative metrics reported in Table I, which suggest solid overall performance in subject segmentation. This combination of visual examples and metrics confirms the model’s robustness in handling:

• age-related anatomical variability (e.g., neonates vs. toddlers),
• diverse patient positions (e.g., supine, curled up),
• and real-world noise due to occlusions and thermographic limitations.

Such adaptability is crucial for deployment in clinical pediatric settings, where patient size, posture, and context vary widely from one case to another.

### D. Heat Segmentation and Occlusion Handling

Thermal images often contain occlusions such as blankets, bandages, or medical devices, which lower the apparent temperature due to their insulating properties. To address this, we implemented a heat-based segmentation stage to isolate bare-skin regions for more accurate energy estimation.

Our approach is based on the physiological hypothesis that exposed skin appears warmer than covered regions in infrared imagery. We apply a Kmeans clustering algorithm to the temperature values within the segmented patient mask, treating temperature as the sole feature. The warmest cluster was assumed to correspond to bare-skin surfaces. This method allows for adaptive separation without requiring manually tuned temperature thresholds.

Unlike simple thresholding, Kmeans clustering adapts to the scene’s thermal variability and helps reduce false positives caused by thermally conductive objects such as bedding or pillows. The result is a binary mask highlighting high-temperature zones suitable for radiative computation.

Additionally, occluded regions are modeled rather than discarded entirely. Based on prior work [6], garments and bandages can be treated as thermal resistances. Under this model, the true skin temperature beneath a textile layer can be recovered via:

\begin{equation*}
 T_{\mathrm{skin}} = T_{\mathrm{cloth}} + \varphi _{\mathrm{occl}} \cdot R_{t} \tag{1}
\end{equation*}

where $T_{\mathrm{cloth}}$ is the temperature measured on the occluding surface, $\varphi _{\mathrm{occl}}$ is the estimated surface heat flux (in W/m$^{2}$), and $R_{t}$ is the thermal resistance of the material (in K$\cdot$ m$^{2}$/W). This model takes into account real-world scenarios with frequent partial occlusions such as blankets, clothing, or medical bandages.

To obtain $R_{t}$ for each textile or bandage type, controlled steady-state heat–transfer measurements were conducted. Each material sample was placed on a temperature-controlled heating plate simulating human skin, while ambient conditions were kept stable. After reaching steady state, the temperature drop across the textile and the heat flux passing through it were recorded. The thermal resistance was subsequently computed as:

\begin{equation*}
 R_{t} = \frac{\Delta T \cdot A_{matetial}}{Q}, \tag{2}
\end{equation*}

where $\Delta T$ (in K) is the temperature difference between the plate and the outer textile surface, $A_{matetial}$ (in m$^{2}$) is the sample area, and $Q$ (in W) is the measured heat flux. This procedure yields material-specific $R_{t}$ values that can be used in (1) to recover the underlying skin temperature.

### E. Radiative Energy Computation

Once bare-skin regions are identified, we compute the radiative energy emitted using the Stefan–Boltzmann law for net thermal radiation [10]:

\begin{equation*}
 P = \varepsilon \sigma \left(T^{4} - T_{\mathrm{ambient}}^{4} \right), \tag{3}
\end{equation*}

where $P$ is the radiative power per unit area ($\mathrm{W/m^{2}}$), $\epsilon$ is the emissivity of human skin (0.98) [11], $\sigma = 5.673 \cdot 10^{-8}$ W $\cdot$K $^{4}\cdot$ m$^{2}$ is the Stefan–Boltzmann constant, $T$ is the mean skin temperature in Kelvin, and $T_{\mathrm{ambient}}$ is the room temperature.

### F. Total Energy Expenditure Estimation

The computed radiative power is integrated over 24 hours to obtain the total emitted energy. To normalize it per unit area, we estimate the patient’s body surface area (BSA) using a pediatric-specific empirical formula:

\begin{equation*}
 A = \frac{4W + 7}{W + 90}, \tag{4}
\end{equation*}

where $W$ is the patient’s weight in kilograms. This formula is widely used in pediatric contexts and has been test for accuracy in children of various ages [12].

Finally, we approximate the TEE by assuming that thermal radiation accounts for approximately 75% of the total daily energy loss in resting ill children [2], [11]:

\begin{equation*}
 \text{TEE (kcal/m}^{2}\mathrm{/day)} = \frac{ P \cdot 86400}{4184 \cdot 0.75}, \tag{5}
\end{equation*}

where 86400 is the number of seconds in a day, and 4184 is the conversion factor from Joules to kilocalories.

### G. Development of THERMIS: An Application for Automated TEE Estimation From Thermography Images

We developed *THERMIS*, a standalone desktop application that consolidates the segmentation and TEE estimation pipeline into a practical and user-friendly platform designed for clinical environments.

*THERMIS* was implemented in Python with a graphical interface built using CustomTkinter. The application integrates the following core functionalities:

• *Data I/O:* import of thermographic images and temperature matrices, and export of results in standardized Excel reports;
• *Segmentation:* automatic extraction of patient regions using the trained U-Net model to isolate relevant anatomical areas;
• *TEE Computation:* estimation of radiative heat losses and calculation of daily total energy expenditure based on thermographic data;
• *Graphical User Interface (GUI):* interactive visualization of input images, segmentation masks, and TEE results, providing accessible feedback for clinical staff.

All computations are performed locally and offline, ensuring reproducibility of analyses and robustness across clinical settings.

### H. Statistical Analysis

The statistical analysis was designed to evaluate both the distribution of estimated TEE values and their relationship with patient age.

The normality of the data was assessed using the Shapiro–Wilk test, which is recommended for small to medium sample sizes due to its sensitivity to deviations from normality. To compare the cohort mean with the clinical reference value of 1500 kcal/m$^{2}$/day [13], a one-sample t-test was performed.

Associations between age and TEE were investigated through correlation analyses. Pearson’s correlation coefficient was applied to measure linear relationships, whereas Spearman’s rank correlation was employed to capture monotonic trends that may not be strictly linear.

This combination of statistical tests was selected to ensure robustness and interpretability with the sample size, while directly addressing the study objectives of assessing distributional properties, comparing means, and evaluating correlations.

## III. Results

### A. Demographics

The dataset for TEE Estimation included 116 pediatric patients. Demographic characteristics are summarized in Table II.

**TABLE II:** Demographic Characteristics of the Patients (N = 116)

**Variable**	**Value**
Number of patients	116
Age (years)	2.3 $\pm$ 3.6 (0.0 – 16.6)
Sex (Female)	43.1%
Weight (kg)	11.5 $\pm$ 10.6 (2.4 – 55.0)
TEE (kcal/m$^{2}$/day)	1547 $\pm$ 279 (757 – 2189)

### B. TEE Distribution and Relationship With Age

**Fig. 4.**
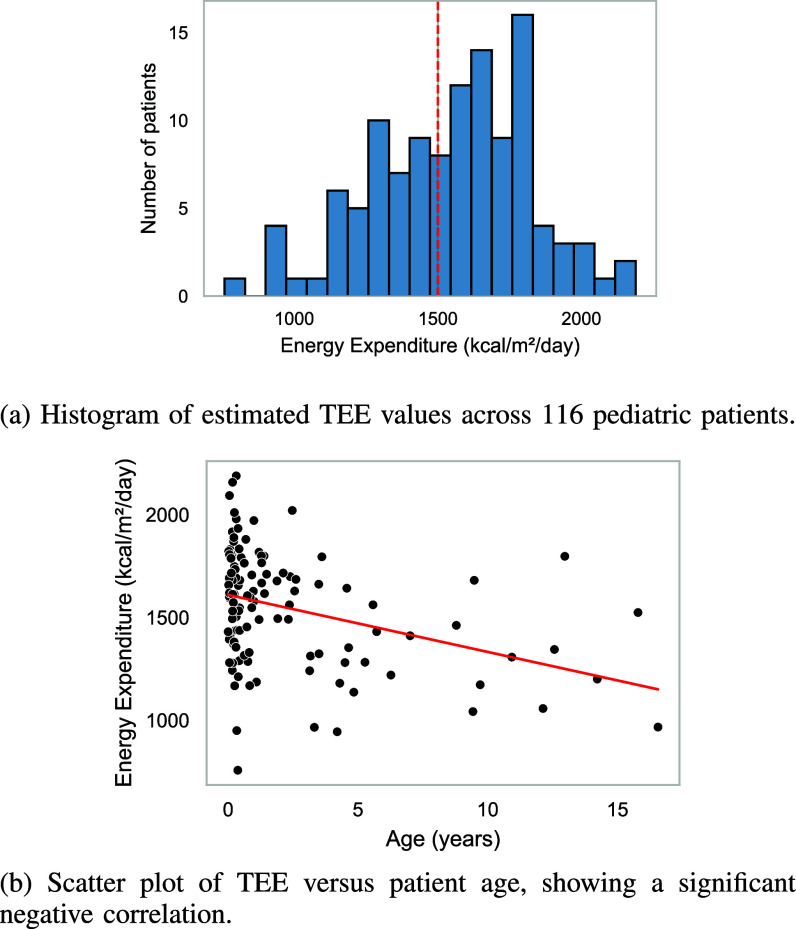
Distribution of estimated TEE and its relationship with patient age. Statistical analyses confirmed normality of TEE values and revealed a significant negative association with age. One-sample t-test comparing the mean TEE to the clinical reference value of 1500 kcal/m$^{2}$/day showed no significant difference ($t = 1.797$, $p = 0.0750$).

Figure 4 summarizes the distribution of estimated TEE across 116 pediatric patients and its relationship with age. Statistical analyses confirmed that TEE values did not significantly deviate from normality (Shapiro–Wilk test, $p = 0.4329$). A one-sample t-test comparing the mean TEE to the clinical reference value of 1500kcal/m$^{2}$/day showed no significant difference ($t = 1.797$, $p = 0.0750$). Correlation analyses revealed a significant negative association between age and TEE (Pearson’s $r = -0.36$, $p = 0.0001$; Spearman’s $r_{s} = -0.34$, $p = 0.0002$), indicating decreasing energy expenditure with increasing age.

### C. Design of the THERMIS Application

**Fig. 5.**
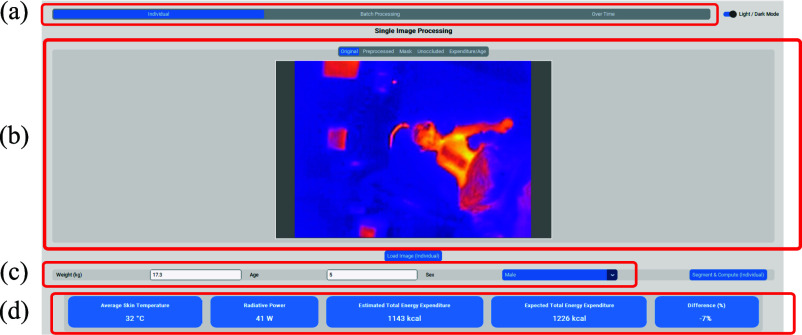
Screenshot of the THERMIS application interface illustrating the four main components: (a) mode selection, (b) image display, (c) patient input fields, and (d) analysis results.

The desktop application THERMIS provides a GUI that integrates image processing, segmentation, and TEE estimation into a single platform for clinical use. Figure 5 shows the main interface with four functional components:

a) *Mode selection:* users can choose between individual image analysis, batch processing of multiple images, or longitudinal monitoring.
b) *Image display:* for each patient, the following are displayed from left to right: the original thermal image, the pre-processed grayscale image, the segmentation mask of the patient, the refined mask excluding occlusions (tubes, dressings), and an indicative TEE estimate compared with reference values by age and sex.
c) *Input fields:* weight, age, and sex of the patient, required for the computation of TEE.
d) *Analysis results:* from left to right, the average skin temperature ($^{\circ }$C), estimated radiative power (W), total caloric expenditure (kcal/day), expected TEE for an individual of the same age and sex, and the percentage deviation from the expected value.

**Fig. 6.**
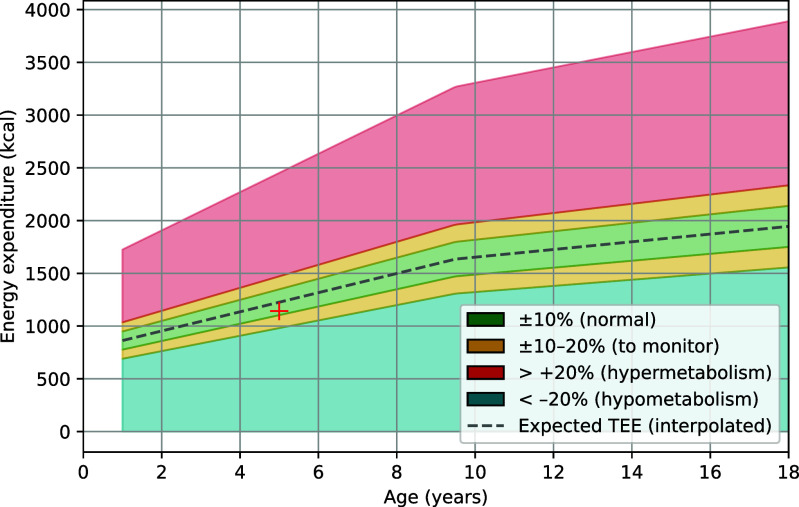
Indicative visualization of patient energy expenditure relative to reference values by age and sex. The cross represents the patient’s TEE, while colored zones indicate ranges of normal metabolism (green), metabolism to monitor (yellow), hypermetabolism (red), and hypometabolism (blue).

In addition, Fig. 6 illustrates the indicative TEE estimate by age and sex, also available in the app’s Expenditure/Age section, as supportive feedback rather than a diagnostic tool.

## IV. Discussion

The primary objective of this study was to develop and test a thermal imaging pipeline designed to estimate energy expenditure in pediatric patients. The analysis was conducted on a dataset of 116 patients, covering a wide range of ages (0–16 years) and clinical conditions.

We adopted the reference value of 1500 kcal/m$^{2}$/day, which is widely used in pediatric clinical nutrition and serves as a standard benchmark for infants and young children, as documented in the educational literature [13]. Thus results obtained from our automated pipeline are consistent: the mean energy expenditure was $1547\pm 279$ kcal/m$^{2}$/day, closely aligning with the widely cited threshold of 1500 kcal/m$^{2}$/day. A one-sample *t*-test confirmed no significant deviation from this reference ($p = 0.075$), suggesting that the pipeline can provide a good estimation of energy expenditure in a heterogeneous pediatric population.

Furthermore, the observed negative correlation between age and energy expenditure (Pearson $r = -0.360$, Spearman $r = -0.343$) is consistent with established physiological trends [14], [15], demonstrating the model’s ability to extract meaningful information from thermal data.

However, in this study the accuracy of TEE measurement using InfraRed Thermal images was not demonstrated. This will be assessed in a clinical trial in 2026 that is already approved by the review ethic board of Ste-Justine (number 2026-9275).

In addition, to consolidate these findings and ensure reproducibility, future studies should aim to replicate the key steps of our work:

1) Curating and annotating a dedicated thermal imaging dataset.
2) Training a robust segmentation model adapted to pediatric thermography.
3) Implementing and testing a TEE estimation framework based on radiative heat transfer principles

In addition, integrating multimodal data sources, such as clinical parameters or metabolic indicators, could further enhance predictive accuracy and clinical relevance.

*Validation Objective:* These findings do not aim to establish new clinical knowledge, but rather to test the methodological performance of the image-based pipeline. The close agreement with reference values and the reproduction of known physiological trends demonstrate the pipeline’s potential for further research and clinical use.

*Limitations and Perspectives:* This study relies on retrospective data and does not control for all confounding variables such as nutritional status or body composition. The thermal images used are of relatively low resolution (160×120 pixels), which may limit segmentation. The temperature estimation accuracy is also dependent on the calibration of the sensor which needs to be more accurate than the automatic calibration used in this study.

A further limitation concerns the assumptions made in converting thermal data to physiological variables. Skin emissivity is assumed constant (0.98), but it can vary with age, pigmentation, hydration, clinical conditions, or the presence of creams, antiseptics, moisture, or dressings, potentially introducing errors. Likewise, assuming that 75% of heat loss occurs via radiation simplifies the energy balance, but this fraction can change during fever, sweating, shivering, or circulatory disorders, and is influenced by airflow and ambient humidity. These factors add uncertainty to temperature-based estimations, suggesting that more refined thermophysiological models could improve accuracy.

Future work may explore deep learning methods to enhance thermal image quality. In particular, super-resolution techniques could upscale and denoise frames, potentially improving segmentation and temperature estimation. Generative adversarial networks (GANs) and convolutional super-resolution networks are promising approaches in this context [16], [17].

## V. Conclusion

The proposed thermal analysis pipeline successfully reproduces expected patterns of energy expenditure in a pediatric population. The alignment with clinical reference values and age-related metabolic trends supports to conduct a clinical study to validate the measurements in comparison with a gold standard such as indirect calorimetry. This work provides a solid foundation for further development, including the integration of image enhancement techniques to improve performance in real-world clinical scenarios.

## Appendix

This appendix provides detailed performance metrics for all tested configurations of the U-Net and DeepLabV3 architectures with a ResNet-50 backbone. Hyperparameters explored included learning rate (lr) and batch size (bs). Metrics include Mean Intersection-over-Union (IoU), Dice coefficient, and Pixel Accuracy.

The best U-Net configuration slightly outperforms the best DeepLabV3 model. This can be explained by U-Net’s ability to perform well on smaller datasets. Additionally, U-Net was preferred for its simpler encoder-decoder architecture.

**TABLE III:** Segmentation Performance for U-Net and DeepLabV3 Architectures With Identical Hyperparameters (Sorted by Mean IoU)

**Architecture**	**lr**	**bs**	**Mean IoU**	**Dice**	**Pixel Accuracy**
U-Net	1e-4	19	0.7530	0.8438	0.9849
U-Net	1e-4	8	0.7519	0.8407	0.9847
U-Net	1e-4	18	0.7180	0.8171	0.9829
U-Net	1e-4	15	0.6959	0.8058	0.9812
U-Net	1e-3	19	0.6928	0.7969	0.9797
U-Net	1e-4	32	0.6651	0.7765	0.9796
U-Net	1e-3	16	0.6526	0.7651	0.9767
U-Net	1e-3	18	0.6460	0.7616	0.9737
U-Net	1e-4	16	0.6342	0.7521	0.9738
U-Net	1e-3	32	0.6276	0.7476	0.9713
U-Net	1e-3	17	0.6251	0.7460	0.9726
U-Net	1e-2	19	0.5599	0.6855	0.9686
U-Net	1e-2	15	0.5397	0.6590	0.9689
DeepLabV3	1e-4	17	0.7484	0.8370	0.9865
DeepLabV3	1e-4	8	0.7316	0.8273	0.9845
DeepLabV3	1e-4	18	0.7414	0.8345	0.9853
DeepLabV3	1e-4	15	0.0520	0.0864	0.9383
DeepLabV3	1e-3	19	0.6557	0.7698	0.9777
DeepLabV3	1e-4	32	0.6491	0.7656	0.9778
DeepLabV3	1e-3	16	0.6376	0.7607	0.9760
DeepLabV3	1e-3	18	0.5721	0.7057	0.9683
DeepLabV3	1e-4	16	0.7089	0.8109	0.9834
DeepLabV3	1e-3	32	0.7041	0.8113	0.9816
DeepLabV3	1e-3	17	0.6376	0.7446	0.9791
DeepLabV3	1e-2	19	0.5430	0.6763	0.9648
DeepLabV3	1e-2	15	0.5291	0.6431	0.9735
